# Induction of Humoral and Cellular Immunity by Intradermal Delivery of SARS-CoV-2 Nucleocapsid Protein Using Dissolvable Microneedles

**DOI:** 10.1155/2021/5531220

**Published:** 2021-05-17

**Authors:** Chaiyaporn Kuwentrai, Jinming Yu, Bao-zhong Zhang, Ye-fan Hu, Ying Dou, Hua-rui Gong, Jian-Dong Huang, Chenjie Xu

**Affiliations:** ^1^School of Biomedical Sciences, Li Ka Shing Faculty of Medicine, The University of Hong Kong, 3/F, Laboratory Block, 21 Sassoon Road, Hong Kong SAR, China; ^2^Department of Biomedical Engineering, City University of Hong Kong, 83 Tat Chee Avenue, Kowloon, Hong Kong SAR, China; ^3^CAS Key Laboratory of Quantitative Engineering Biology, Shenzhen Institute of Synthetic Biology, Shenzhen Institutes of Advanced Technology, Chinese Academy of Sciences, Shenzhen 518055, China; ^4^Department of Medicine, University of Hong Kong, 4/F Professional Block, Queen Mary Hospital, 102 Pokfulam Road, Hong Kong SAR, China

## Abstract

The nucleocapsid protein (NP) of the severe acute respiratory syndrome coronavirus 2 (SARS-CoV-2) contains immunogenic epitopes that can induce cytotoxic T lymphocyte (CTL) against viral infection. This makes the nucleocapsid protein a suitable candidate for developing a vaccine against SARS-CoV-2 infection. This article reports the intradermal delivery of NP antigen using dissolvable microneedle skin patches that could induce both significant B cell and T cell responses.

## 1. Introduction

The severe acute respiratory syndrome coronavirus 2 (SARS-CoV-2) has triggered a global pandemic with extremely rapid infection [1–3]. Candidate vaccines are being tested for their ability to prevent infection/reinfection of SARS-CoV-2. Currently, there are several promising spike-based vaccines from different developers such as Pfizer/BioNTech and Sinovac, but these vaccines have yet achieved sterilizing immunity in the population. Furthermore, there is limited information on how long the protective immunity could last, especially when variants of SARS-CoV-2 are emerging constantly. Single-antigen vaccines may easily lose their protection effect due to the mutations. Therefore, our group aims to investigate nonspike vaccine candidates that may induce additional, long-lasting or more complete protective responses in patients. Apart from the conventional SARS-CoV-2 spike glycoprotein (S protein), the nucleocapsid protein (NP) has been identified as an alternative candidate for vaccine development due to its ability to induce antiviral cytotoxic T cell responses [4]. NP plays a vital role in viral host cell entry and modulates virus particle assemble and release [5]. Notably, there are many human leukocyte antigen (HLA) binding peptides within NP, which could prime specific CD8+ and CD4+ T cell responses via major histocompatibility complex class 1 (MHC1) and major histocompatibility complex class 2 (MHC2) pathways, respectively [6]. Furthermore, immunoblot assays have revealed the presence of IgA, IgM, and IgG antibodies against N antigen in COVID-19 infection patients' sera [7]. More recently, Chukwudozie et al. developed a subcomponent vaccine targeting the SARS-CoV-2 nucleocapsid phosphoprotein RNA binding domain. This vaccine contains both antigenic B cell and T cell epitopes that provide a sufficient antigenic index and nonallergenic property [8]. Importantly, Peng et al. revealed that CD8+ T cell responses were associated with milder disease (2-3 weeks recovery rates), whereas more severe cases of SARS-CoV-2 had lower CD8+ T cell responses. Typically, it only takes 7 to 10 days for COVID-19 virus infections to activate antiviral T cell responses which correlate well with the time taken for milder disease patients to recover. This indicates that T cell immune responses play an important role in the prevention of SARs-COV-2 pathogenesis and possibly longer-term protective immunity [9]. The pathogenesis of SARS-CoV-2 involves impaired dendritic cell responses along with delayed T cell responses. These delayed T cell responses exhibited greater CD4+ T cells than CD8+ T cells [10]. Collectively, these preliminary evidences support the prospect of developing alternative nonspike vaccine candidates, such as NP, that could elicit specific T cell responses against SARS-CoV-2. Our group aims to investigate microneedle-delivered NP vaccines that may induce additional, long-lasting or more complete protective responses in patients.

The storage, transport, and administration of the SARS-CoV-2 vaccine are vital considerations for vaccine development and control of the pandemic worldwide. The majority of approved SARS-CoV-2 vaccines, such as the Pfizer-BioNTech BNT162b2 vaccine or Moderna's mRNA-1273 vaccine, are administered by intramuscular (IM) injection. However, such conventional injection techniques may induce the risk of blood-related infections and require trained personnel to perform injections [11–15]. These constraints may be solved by other nonconventional techniques.

Microneedle- (MN-) based intradermal delivery offers unique benefits, such as reduced biohazard waste, stable storage at room temperature, ease of distribution, and pain-free vaccination. The rationale behind MN delivery of antigen vaccines into the skin is to activate the adaptive immune response via antigen-presenting dendritic cells, Langerhans cells, and other cells that could prime antigen-specific immunity [16]. There has also been examples of MN devices that could effectively deliver SARS-CoV-2 spike subunit vaccines to the mice skin, such as Kim's and Kuwentrai's work [17, 18]. Encouraged by these research findings, our current study investigates the potential use of dissolvable MNs for the intradermal delivery of NP as an alternative vaccine for COVID-19. Uniquely, our MN device is composed of NP and low-molecular weight (48 K) hyaluronic acid (HA) that is formulated by the micromolding method [19–23]. In the mouse model, NP-MNs elicit significant B cell antibody responses and interferon-gamma- (IFN-*γ-*) based T cell responses compared to nonimmunized controls. Lung immunohistochemical analysis is used to examine T cell markers (CD4 and CD8) in MN vaccinated mice that might play a protective role against respiratory tract coronavirus infections [24, 25].

## 2. Materials and Methods

### 2.1. Nucleocapsid Protein Preparation

NP of SARS-CoV-2 (418 amino acids) was cloned and purified from *E. coli* as previously reported [26]. This protein was mixed at a ratio of 9 : 1 with aluminum hydroxide gel (InvivoGen).

### 2.2. MN Fabrication

MN patches were produced through the micromolding method. Briefly, HA (molecular weight: 48 K, 100 mg/mL) was dissolved in deionized water (100 mg/mL). NP (25 *μ*g) formulated with aluminum hydroxide gel was vortexed with the HA solution. Next, 50 *μ*L of the mixture was added to a polymethylsiloxane (PDMS) negative mold and centrifuged at 4,000 rpm for 3 minutes to make sure that all cavities in the mold were completely filled. After drying overnight at room temperature, additional HA solution was added to form the backing part of the patch. After drying, the patch was peeled off from the micromold and preserved in a dry box until use.

### 2.3. Animal Experiments

All BALB/c mice were obtained and purchased from the Laboratory Animal Unit of the University of Hong Kong. All animal experiments were approved by the Committee on the Use of Live Animals in Teaching & Research, the University of Hong Kong (#CULATR 5312-20). Vaccination was conducted using intradermal delivery of MN-formulated NP (25 *μ*g/mice) or subcutaneous injection of NP (25 *μ*g/mice) on day 0, 3, and 7. Blood samples were obtained from the tail vein on day 14, 21, and 28.

### 2.4. IFN-*γ* ELISPOT Assay

On day 28, mice were sacrificed, and spleen cells were obtained for IFN-*γ* ELISPOT analysis. Briefly, 100 *μ*L of spleen cells was incubated on the IFN-*γ* ELISPOT plate, which was activated for 30 minutes using 200 *μ*L DMEM media without FBS. Subsequently, 5 *μ*g of the peptide (NP and the positive inducer) was added, and cells were kept for 20 hours at 37°C in 5% CO_2_. After the media was discarded, cells were washed using a washing buffer and incubated with the secondary biotinylated antibody for 1 hour at room temperature (RT). Finally, cells were incubated with the biotin substrate, followed by washing and drying to produce visible spots in positive wells.

### 2.5. Enzyme-Linked Immunosorbent Assay (ELISA)

At day 14, 21, and 28 from the first vaccination, blood obtained from tail vein was centrifuged at 3,000 rpm for 30 minutes. All mice serum was stored at -80°C until further characterization. A 96-well ELISA plate was coated with 10 *μ*g/mL of antigen (NP) in coating medium. Specifically, 100 *μ*L of the solution was added to each well at 4°C and kept overnight. After 12 hours, the solution was discarded, and the plate was blocked by blocking buffer (5% milk in tris-buffered saline mixed with tween 20 [TBST]) for 3 hours at RT. The wells were then washed with TBST six times to remove milk precipitates. Next, serum collected from above was serially diluted in milk-TBST solution at the following ratios: 1 : 3, 1 : 12, 1 : 48, 1 : 192, 1 : 768, 1 : 3072, 1 : 12288, and 1 : 49152. The diluted serum was added to the wells and kept for 1 hour at RT. The plate was washed five times in 1X TBST and then incubated with mouse IgG, mouse IgG1, or mouse IgG2A secondary antibody diluted in milk-TBST solution at a ratio of 1 : 3000. After incubation for 1 hour at RT, the plate was washed six times using 1X TBST. Next, 100 *μ*L of 3,3′,5,5′-tetramethylbenzidine (TMB substrate) was added to each well and kept at 37°C for 30 minutes. Finally, the reaction was stopped with H_2_SO_4_ (50 *μ*L per well), and the absorbance collectively was read at 450 nm. ELISA data were obtained on a Varioskan Flash spectral scanning multimode reader (Thermo Scientific).

### 2.6. Cryosectioning and Confocal Microscopy

After mice were sacrificed, lung tissues were harvested and fix overnight in 4% PFA at 4°C with gentle shaking. Tissues were washed three times for five minutes with PBS and transferred to 30% sucrose-solution (30 g Sucrose per 100 mL PBS). Optimal cutting temperature (OCT) compound was added in labeled cryomold and embed tissue, and cryostat sections were cut at 15-20 *μ*m using Cryostar machine and mounted on gelatine-coated histological slides. Section slides were treated with anti-CD4 (1 : 50) and CD8 (1 : 50) mouse primary antibodies (Invitrogen) diluted in blocking buffer and incubated overnight followed by addition of Alexa488-conjugated anti-rat secondary antibodies. Sections were mounted with DAPI-infused mounting media and visualized using a fluorescence confocal microscope at ×20 (LSM 800).

### 2.7. Statistical Analysis

All results were plotted in Prism 7 (GraphPad Software Inc., CA). Statistical comparisons between groups for ELISA were analyzed by the unpaired nonparametric *t*-test (Mann–Whitney test) using Prism 7. Statistical comparisons between groups for ELISPOT were analyzed by the unpaired parametric *t*-test using Prism 7. For all tests, *P* < 0.05 was considered statistically significant.

## 3. Results

### 3.1. Intradermal Delivery of NP Using Dissolvable MNs

**Figure 1:**
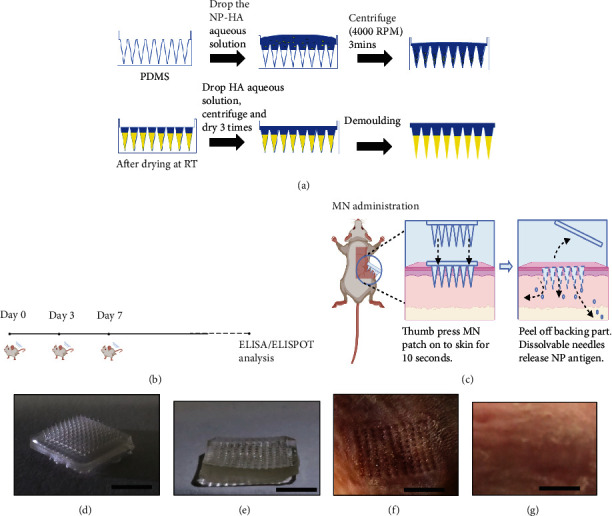
Schematic illustration of the vaccination with MN NPs: (a) MN NP fabrication process, (b) vaccination process, (c) MN NP administration on mice, image of MN NP patch (d) before deployment (tips present) (scale bar, 5 mm) and (e) after deployment (tips absent) (scale bar, 5 mm), and image of the mouse skin (f) immediately after injection (scale bar, 5 mm) and (g) 24 hours after injection (scale bar, 5 mm).

The HA-based MN patches were made by a two-step micromolding method (Figure 1(a)) [27, 28]. Firstly, the mixture of NP and HA filled the MN tip cavities in the PDMS negative mold. Then, the backing of MNs was made using the blank HA solution. We loaded each patch (1 × 1 cm^2^ with 100 MN tips) with ~25 *μ*g NP, which was determined in the previous study [26]. Control patches were also made without NP. Both NP and control patches were thumb pressed into the shaved skin of BALB/c mice at days 0, 3, and 7 (*n* = 5 per group) (Figures 1(b) and 1(c)). 10 seconds postinsertion, the MN tips detached from the base (Figures 1(d) and 1(e)). There were MN marks right after the application, which disappeared after 24 hours (Figures 1(f) and 1(g)).

### 3.2. MN-Delivered NP Induced Specific B Cell Antibody Responses

**Figure 2:**
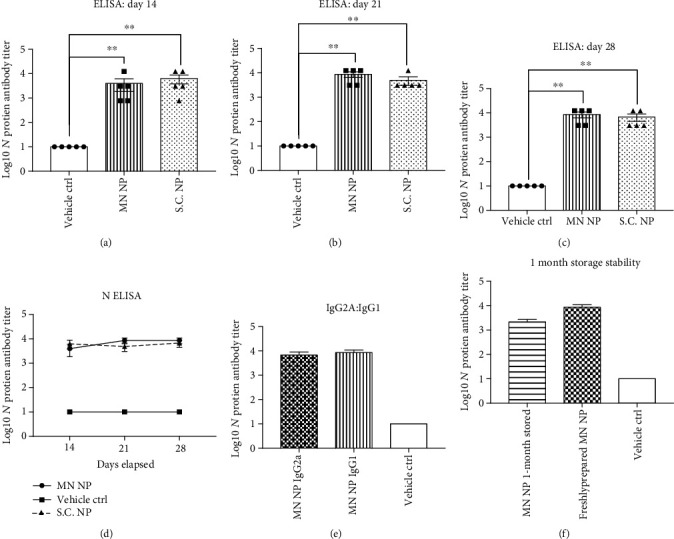
Specific B cell antibody responses to vaccination. ELISA results of serum at (a) day 14, (b) day 21, and (c) day 28 postvaccination, showing log10 antibody titers against the N protein in the S.C. NP immunization group, MN NP immunization group, and vehicle-control group. (d) Summary of log10 mean NP antibody titers from S.C. NP, MN NP, and vehicle-control MN groups. (e) ELISA results of serum at day 28 postvaccination, showing log10 IgG1 and IgG2A antibody titers against the N protein in the MN NP immunization group. (f) ELISA results of mice serum at day 28 postvaccination, showing log10 antibody titers against NP in the freshly prepared MN NP immunization group, 1 month stored MN NP immunization group and vehicle-control group. ELISA absorbance measurements at 450 nm were normalized to standard cut-off values. Student's unpaired nonparametric *t*-test (Mann–Whitney) was used with multiple *t*-test adjustment. Data were expressed as mean ± SEM. ^∗^*P* < 0.05, ^∗∗^*P* < 0.01, ^∗∗∗^*P* < 0.001, ^∗∗∗∗^*P* < 0.0001, ns refers to “not significant”.

The specific B cell antibody response post the delivery of NP was studied through the ELISA assay of IgG antibodies in sera against SARS-CoV-2. There were three groups including subcutaneously injected (S.C.) NP (25 *μ*g/mice), MN-delivered NP (25 *μ*g/mice), or MN-delivered vehicle controls. There was no significant difference between the S.C. NP group and the MN NP group (Figures 2(a)–2(d)). Both groups showed strong immune response. NP antibody titers could be seen at day 14 after the first immunization, which elevated at day 21, and further increased to a maximum antibody titer of 1.2 × 10^4^ at day 28. By day 28, an average antibody titer of over 8.6 × 10^3^ was detected, indicating that both the MN and S.C. delivery of NP were highly effective. In comparison, there was no comparable immune response in the control group. Importantly, the MN NP group exhibited a balanced ratio of IgG1 and IgG2A NP-specific antibodies, which is vital for viral clearance (Figure 2(e)). Furthermore, MN NP patches that were stored in a dehumidifier (25 degrees) induced comparable NP-specific antibodies in mice compared to freshly prepared MN NP patches at day 28 after immunization (Figure 2(f)).

### 3.3. MN-Delivered NP Elicited Specific T Cell Responses

**Figure 3:**
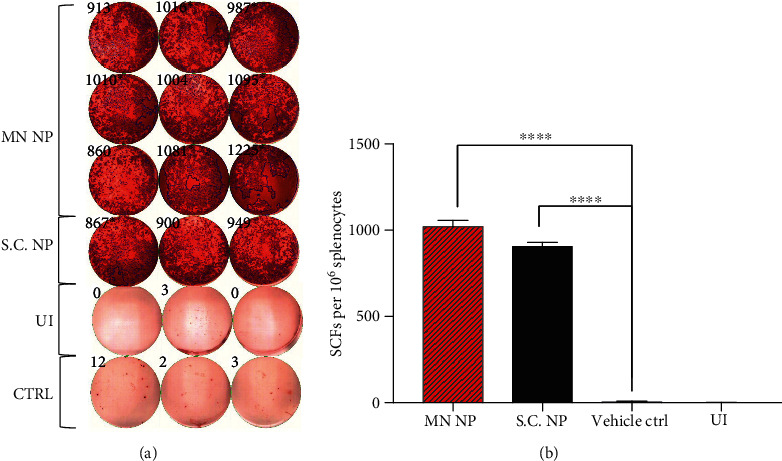
IFN-*γ* T cell responses. (a) IFN-*γ* ELISPOT plate counts for S.C. NP stimulated, MN NP stimulated, blank MN stimulated (CTRL), and nonstimulated (UI) groups. (b) Bar chart shows mean IFN-*γ* ELISPOT counts for all four groups. Student's unpaired parametric *t*-test was used. Data were expressed as mean ± SD. ^∗^*P* < 0.05, ^∗∗^*P* < 0.01, ^∗∗∗^*P* < 0.001, ^∗∗∗∗^*P* < 0.0001, ns refers to “not significant”.

We investigated the T cell IFN-*γ* response in mouse splenocyte immunization by S.C. NP, MN NP, and vehicle-control MN by using ELISPOT with the NP protein (5 *μ*g/mL) as the stimulator. Ionomycin was used as the positive inducer, and FBS-free medium was used as the negative inducer. As shown in Figures 3(a) and 3(b), significant IFN-*γ* was released by T cells by day 29 in the S.C. NP and MN NP groups compared to vehicle and noninduced controls. The elevated levels of IFN-*γ* producing T cells may produce strong antiviral protection against SARS-CoV-2.

### 3.4. Immunohistochemical Staining of T Cell Markers within Mouse Lung Tissues Posts the Stimulation with MN-Delivered NP

**Figure 4:**
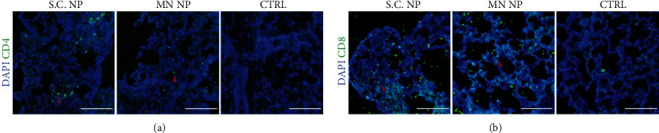
Immunohistochemical staining of lung tissues from MN-based NP immunized mice. At day 29, mice from the S.C. NP immunization group, MN NP immunization group, and vehicle control groups were sacrificed and lung tissues were collected and subjected to IHC staining of CD4 (a) and CD8 (b) markers. Sections were viewed under LSM800 confocal microscope. Scale bars, 100 *μ*m.

Zhao et al. showed that Venezuelan equine encephalitis replicons (VRP) SARS-CoV NP induced CD4+ T cells in the lungs, which played an important role in SARS-CoV and MERS-CoV protection [24]. Habel et al. also showed that CD8+ T cell responses in the lungs were critical for COVID-19 recovery [25]. Based on these observations, we examine the presence of CD4 and CD8 T cell markers in the mouse lungs post the delivery of NPs (Figure 4). CD4 staining represents T helper cells, whereas CD8 represents cytotoxic T cells. Both types of immune cells in the lungs are present after the MN and S.C. delivery of NP, which may confer protection towards SARS-CoV-2.

## 4. Discussion and Conclusion

This study reports the successful intradermal delivery of NP using dissolvable HA MNs that could induce specific B cell antibodies and IFN-*γ* T cell responses in mice. This MN formulation is composed of NP antigens, HA, and aluminum hydroxide gel adjuvant.

Previously, Kim et al. delivered recombinant coronavirus vaccines (SARS-CoV-2-S1 and SARS-CoV-2-S1fRS09 subunit) using carboxymethyl cellulose-based MN delivery platforms [17]. Moreover, our team previously explored the use of HA-based MN delivery platform to deliver S-RBD (SARS-CoV-2-S1) in mice and obtained similar results [18]. In this work, we investigated the use of HA-based MN delivery platform to deliver NP in mice. The rationale behind this is to illustrate the immunogenicity of NP as an alternative antigen that could induce specific B and T cell responses in a similar manner to the conventional spike protein SARS-CoV-2 vaccine target.

Our work supports the NP as an alternative vaccine candidate for SARS-CoV-2. We showed that MN NP could trigger significant B cell antibody responses and significant T cell IFN-*γ* responses. Notably, T cell IFN-*γ* responses towards viral antigens have been reported as critical antiviral protective factors that partake in the eradication of COVID-19 [26]. T cell responses may be as vital as the induction of specific B cell antibodies against SARS-CoV-2 [29, 30]. Previous work by Zhao et al. showed that CD4+ T cells in the lungs are induced after intranasal immunization by VRP SARS-CoV nucleocapsid protein, and that these cells play an important role in SARS-CoV and MERS-CoV protection [24]. Habel et al. also recently discovered that CD8+ T cell immunity is vital for virus protection amongst COVID-19 patients. Thus, we performed immunohistochemical staining of lung tissues for MN NP, S.C. NP, and control MN groups to demonstrate presence of CD4 and CD8 T cell markers in the lungs of NP immunized mice. On the other hand, Wang and colleagues discovered that the neutralization activity of spike-targeting mRNA vaccines decreased against the mutated COVID-19 strain, 501Y.V2 [31]. Furthermore, a recent study by Li et al. demonstrated the effects of different mutations on the spike that could hinder the effects of neutralizing antibodies against the antigen. For instance, the N234Q mutation decreased neutralization sensitivity against different monoclonal antibodies. Whether these mutations have a direct effect towards protective vaccine efficacy remains to be investigated [32]. These findings support the development of additional nonspike candidates, such as NP, to fully eradicate new mutated strains. On the other hand, there are emerging clinical studies about combining different treatments. For instance, Hung et al. administered either a 14-day combination of lopinavir 400 mg and ritonavir 100 mg every 12 h, ribavirin 400 mg every 12 h, and three doses of 8 million international units of interferon beta-1b on alternate days (combination group) or to 14 days of lopinavir 400 mg and ritonavir 100 mg every 12 h (control group) to randomly assigned COVID-19 patients in a phase 2 trial [33]. Therefore, another consideration is to combine NP vaccines with spike vaccines to augment efficacy. Interestingly, we also discovered that it is possible to combine 2 proteins into a single microneedle (supplementary Figure S1).

The MN-based intradermal delivery is a unique method for administering vaccines with distinct benefits such as noninvasiveness, full biocompatibility, fast vaccination deployment, sustained drug release, ease of use, and reduction of logistic costs and wastage. In our work, we used dissolvable HA MNs to administer NP vaccines, allowing sustained drug release into tissue layers beneath the skin for triggering adaptive immunity. The sustained release of NP vaccines increases drug utility by reducing fluctuations in steady-state drug levels and enhances the safety margin of the drug activity [34]. Microneedle skin patches (area per patch = 10 mm × 10mm, individual needle length = 1 mm) may be administered quickly by thumb pressing on to the skin for at least 10 seconds before peeling. The fast and straight-forward administration process of MN devices dominates over conventional S.C. procedures that require trained personnel to accurately inject the vaccine underneath the skin. As mentioned in our manuscript, microneedle penetration of the mice skin led to penetration marks indicating successful delivery of microneedle components (Figure 1(f)). We found that these penetration marks disappeared overnight after administration (Figure 1(g)). There was lack of scarring on the mice skin which also supports the noninvasive nature of MN vaccine delivery. Our MN devices are formulated using biodegradable 50 kDa hyaluronic acid (HA) which is a well-studied substance known for its natural occurrence in the skin, good solubility, and lack of reported side effects. The biodegradable nature of MN vaccines prevents the requirement for safe needle disposal and waste, unlike for S.C. injections. Furthermore, preformulated MN vaccines can be stored at room temperature which promotes vaccine storage and distribution in developing countries with limited cold chain. Alternatively, several factors may limit the efficiency of the MN delivery system for NP, such as possible loss of antigen activity during the MN formulation and storage. However, we found that the titers of specific NP antibodies produced by the MN method were comparable to the S.C. injection method (Figure 2). The NP-specific IgG2A and IgG1 titers induced by the MN NP group were also comparable, indicating a balanced Th2 and Th1 immunity. Additionally, 1 month's storage of MN NP in a dehumidifier does not seem to affect the activity of inducing NP specific antibodies in mice compared to freshly prepared patches (Figure 2).

In the next developmental stage, the clinical translation of MN vaccine technology still requires the optimization several other key factors. These factors include ensuring device sterility within germ-free production laboratory sites, promoting cost-effective fabrication in large scale, and developing applicators for consistent deployment of MNs. We hope to address these factors in our future work to optimize the HA MN delivery for clinical testing of human COVID-19 patients. For our future work, we also plan to conduct further clinical investigations on the dosing regimen required for combining MN NP with MN S-RBD as a combination therapy regimen for COVID-19 patients.

## Data Availability

The data that support the findings of this study are available from the corresponding author upon request.

## Supplementary Materials

Supplementary Materials We have included one additional supplementary file containing the graphical abstract and Figure S1.
