# Supplementary material for: Induction of Humoral and Cellular Immunity by Intradermal Delivery of SARS-CoV-2 Nucleocapsid Protein Using Dissolvable Microneedles
Source: J Immunol Res. 2021 May 17;2021:5531220. doi: 10.1155/2021/5531220 (PMC8130907; doi:10.1155/2021/5531220)
Supplement: Supplementary Materials — We have included one additional supplementary file containing the graphical abstract and Figure S1. [file 5531220.f1.docx]

**Graphical Abstract**


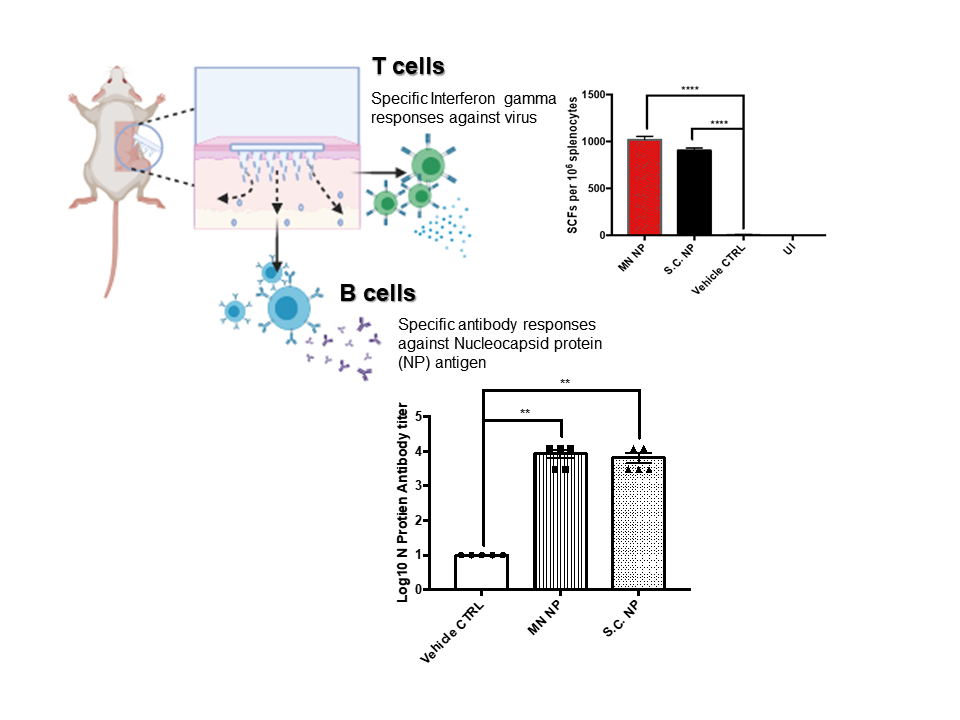


**Figure S1:** Characterization of hyaluronic acid (HA) microneedles (MNs) after loading with two different IgG-tagged proteins. (a) Confocal Image of HA MN tip containing both Alexa Fluor‐555 goat IgG protein (red fluorescence) and Alexa Fluor-488 rat IgG protein (green fluorescence) viewed under green and red fluorescent channels (scale bar, 5 mm). (b) Confocal Image of HA MN tip containing both Alexa Fluor‐555 goat IgG protein (red fluorescence) and Alexa Fluor-488 rat IgG protein (green fluorescence) viewed under green channel only (scale bar, 5 mm). (c) Confocal Image of HA MN tip containing both Alexa Fluor‐555 goat IgG protein (red fluorescence) and Alexa Fluor-488 rat IgG protein (green fluorescence) viewed under red channel only (scale bar, 5 mm).


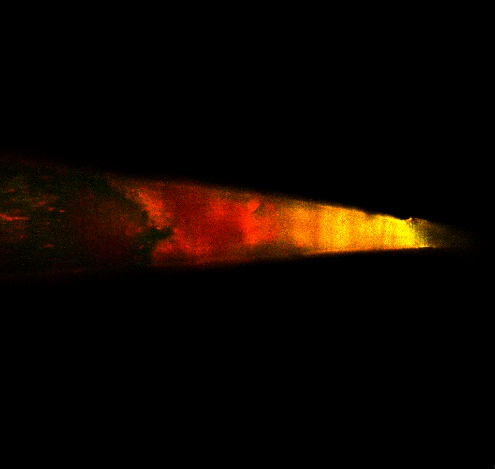


**A**


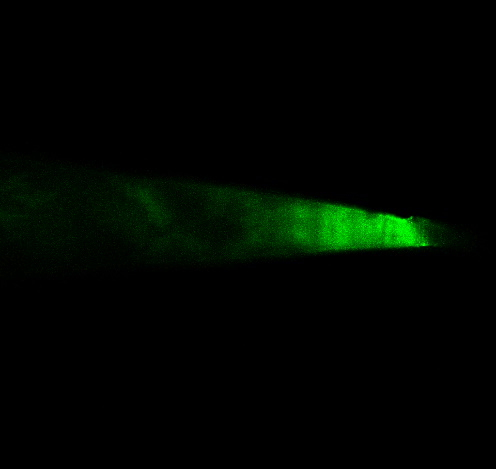


**B**


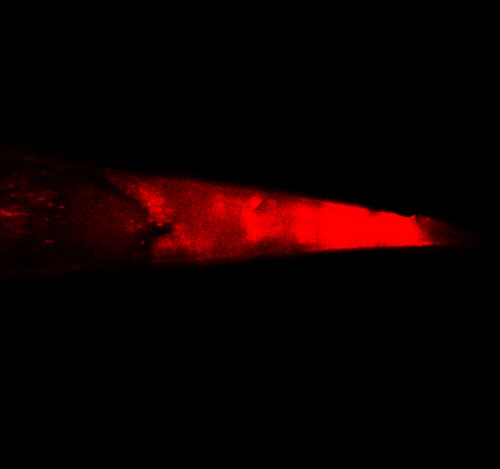


**C**
